# A Predictive Role of Autoantibodies Against the Epitope aa168–183 of ENO1 in the Occurrence of Miscarriage Related to Thyroid Autoimmunity

**DOI:** 10.3389/fimmu.2022.890502

**Published:** 2022-05-30

**Authors:** Xiaoqing He, Yifu Liu, Haoyu Wang, Wei Sun, Yihan Lu, Zhongyan Shan, Weiping Teng, Jing Li

**Affiliations:** Department of Endocrinology and Metabolism, Institute of Endocrinology, NHC Key Laboratory of Diagnosis and Treatment of Thyroid Diseases, The First Affiliated Hospital of China Medical University, Shenyang, China

**Keywords:** α-enolase, autoantibodies, epitope, miscarriage, thyroid autoimmunity

## Abstract

**Objective:**

The aim of the research is to study the association between the serum levels of autoantibodies against one important epitope (^168^FMILPVGAANFREAMR^183^, designated as P6) of α-enolase (ENO1-P6Abs) and miscarriage among euthyroid females with thyroid autoimmunity (TAI).

**Methods:**

Anti-ENO1-P6 total IgG was investigated in 432 euthyroid women, and its four subclasses were analyzed in 184 euthyroid women. The serum FT4, TSH, TgAb, and TPOAb levels were determined using an electrochemiluminescence immunoassay. The serum ENO1-P6Ab and anti-protein disulfide isomerase A3 autoantibody (PDIA3Ab) levels were determined using an enzyme-linked immunosorbent assay.

**Results:**

The serum levels of anti-ENO1-P6 total IgG, IgG2, IgG3, and IgG4 were significantly higher in euthyroid TAI females than in non-TAI controls. Additionally, anti-ENO1-P6 total IgG and its 4 subtypes were all markedly higher in euthyroid TAI females with pregnancy loss than those without miscarriage. Moreover, logistic regression analysis showed that highly expressed anti-ENO1-P6 total IgG, IgG1, IgG2, and IgG3 subtypes in the serum were all independent risk factors for euthyroid TAI-related miscarriage, and its IgG1 was also for non-TAI-related abortion. According to the trend test, the prevalence of miscarriage was increased in a titer-dependent manner with the raised levels of serum anti-ENO1-P6 total IgG and IgG1, IgG2, and IgG3 subtypes among euthyroid TAI females. The receiver operating characteristic curve analysis of anti-ENO1-P6 total IgG and IgG1, IgG2, and IgG3 subclass expressions in the serum for miscarriage prediction in euthyroid TAI females exhibited that the total areas under the curves were 0.773 ± 0.041, 0.761 ± 0.053, 0.827 ± 0.043, and 0.760 ± 0.050, respectively (all *P <*0.0001). Their corresponding optimal cut-off OD450 values were 0.68 (total IgG), 0.26 (IgG1), 0.97 (IgG2), and 0.48 (IgG3), with sensitivities of 70.8, 87.5, 83.3, and 85.4%, and specificities of 70.8, 59.1, 77.3, and 56.8%, respectively. There was an additive interaction between serum anti-ENO1-P6 and anti-PDIA3 total IgGs on the development of miscarriage (RERI = 23.6, AP = 0.79, SI = 5.37).

**Conclusion:**

The highly expressed ENO1-P6Abs may be important risk factors for euthyroid TAI-related miscarriage. The serum levels of ENO1-P6Abs may become good predictive markers for pregnancy loss in euthyroid TAI females, especially its IgG2 subclass expression.

## Introduction

Thyroid autoimmunity (TAI) is the most common autoimmune thyroid disease, which is usually characterized by high expression of anti-thyroperoxidase autoantibody (TPOAb) and anti-thyroglobulin autoantibody (TgAb) and local lymphocyte infiltration. The pathogenesis of the disease is still unclear in which genetic susceptibility, endogenous, and environmental factors work together (1). According to the Thyroid Disease, Iodine status, and Diabetes National Epidemiological (TIDE) survey in 31 Chinese provinces from 2015 to 2017, the prevalence of TAI was 20.35% in women and 8.16% in men (2). It has been found that TAI does not only affect the thyroid but also causes extra-thyroidal injuries (3). Stagnaro-Green et al. have previously reported that 17% of TAI women suffered from spontaneous abortion, whereas only 8.4% of women without TAI had miscarriage (4). Many of the later studies have demonstrated the raised risk of miscarriage in euthyroid TAI women undergoing either natural conception or *in vitro* fertilization (IVF) procedures due to subfertility (5–8). Although not clearly known, the potential mechanisms of spontaneous abortion in TAI women have been attributed to the direct effects of the classical anti-thyroid autoantibodies (ATA), high serum levels of thyrotropin (TSH), cellular immunity, and concurrent non-organ specific autoantibodies (NOSA) (9, 10). There is a lack of direct evidence about the effects of ATA on the occurrence of miscarriage among TAI. Previous studies analyzed the relationship between serum ATA titers and the risk of pregnancy loss in women, but they did not find the presence of titer-dependence (4, 11). The levothyroxine (LT4) treatment of euthyroid TAI women did not increase the live birth rate (12). No significant difference was found in the miscarriage rate between the LT4-treated group and the controls (17.1% *vs.* 19.9%) (13). Anti-protein disulfide isomerase A3 autoantibody (PDIA3Ab) is a NOSA. We have recently found that serum PDIA3Ab expression is independently associated with TAI-related miscarriage (14). It has been noticed that not only one NOSA can contribute to the development of spontaneous abortion in TAI women. Therefore, NOSA in the pathogenesis of pregnancy loss among euthyroid TAI women needs to be focused on.

α-enolase (ENO1) has been known as a multi-functional protein, such as a SUPER molecule on the surfaces of many eukaryotic cells, a c-myc promoter binding protein in the nuclei, and the key glycolytic enzyme in the cytosol (15, 16). The SUPER refers to Surface-exposed (during apoptotic cell death), Ubiquitously expressed, Protease sensitive, Evolutionary-conserved, and Resident normally in viable cells (15). ENO1 protein is widely distributed in various organs, such as the thyroid, brain, placenta, and uterus (17–20). Anti-ENO1 autoantibodies (ENO1Abs) have been found in a few autoimmune and inflammatory disorders (16). Their expression has also been found to be associated with unexplained recurrent miscarriage (uRM), endometriosis, premature ovarian failure, and tubal factor infertility (21–24). In our previous study, the increased serum levels of ENO1Abs were observed in mice with experimental autoimmune thyroiditis (EAT) induced only by Tg immunization (18). Those studies suggest that ENO1Abs may be involved in the development of TAI-related pregnancy loss. There are only a few studies on disease-specific epitopes (DSEs) of ENO1 as an autoantigen, and no investigation has been reported on either miscarriage or TAI. It is necessary to identify the responsible autoantigen epitopes, which can help better understand its pathogenesis and explore new predictive markers and therapeutic targets for the disease.

In the previous pilot study, we identified 18 potential immunodominant epitopes designated as P1–18 through the IEDB, ABCpred, APCpred, BCpred, Bepipred 2.0, DiscoTope2.0, and Ellipro databases. The P6 epitope refers to the amino acid (aa) 168–183 (i.e., FMILPVGAANFREAMR) in the ENO1 protein. The aa153–169 is involved in catalyzing the conversion of 2-phospho-D-glycerate (PGA) to phosphoenolpyruvate (PEP) during the glycolysis process (25). When the aa sequences of ENO1 from different species are compared, the aa168–175 region is highly conserved (25). Thus, we investigated the relationship between the expression of ENO1-P6Abs and the occurrence of pregnancy loss among euthyroid TAI females in this cohort study.

## Materials and Methods

### Study Design and Participants

All the participants in this retrospective investigation were recruited from the Subclinical Hypothyroid in Early Pregnancy (SHEP) study. The SHEP project was a large-scale population-based study involving 19 hospitals in the Liaoning Province of China to study the effects of thyroid abnormalities on pregnancy outcomes and the benefits of LT4 treatments (5, 26–29). The study was approved by the Medical Ethics Committee of China Medical University ([2012]2011-32-4). All participants completed consent forms when enrolled in the trial. All participants were asked to fill out the questionnaires about their personal information on the first visit, which included age, gestational age, alcohol use, smoking, residential city, educational background, family income, and history related to medication, diseases, reproduction, and family members. Height and weight measurements were performed by physicians, and body mass index (BMI) was calculated by weight/height^2^ (kg/m^2^). On the day of enrollment, all individuals underwent an ultrasound examination to confirm the presence of an ongoing intrauterine pregnancy. TAI was confirmed in those pregnant women with raised serum TPOAb (>34 IU/ml) and/or TgAb (>115 IU/ml) levels, for which reference ranges were provided by the manufacturer (Roche Diagnostics, Switzerland).

**Figure 1:**
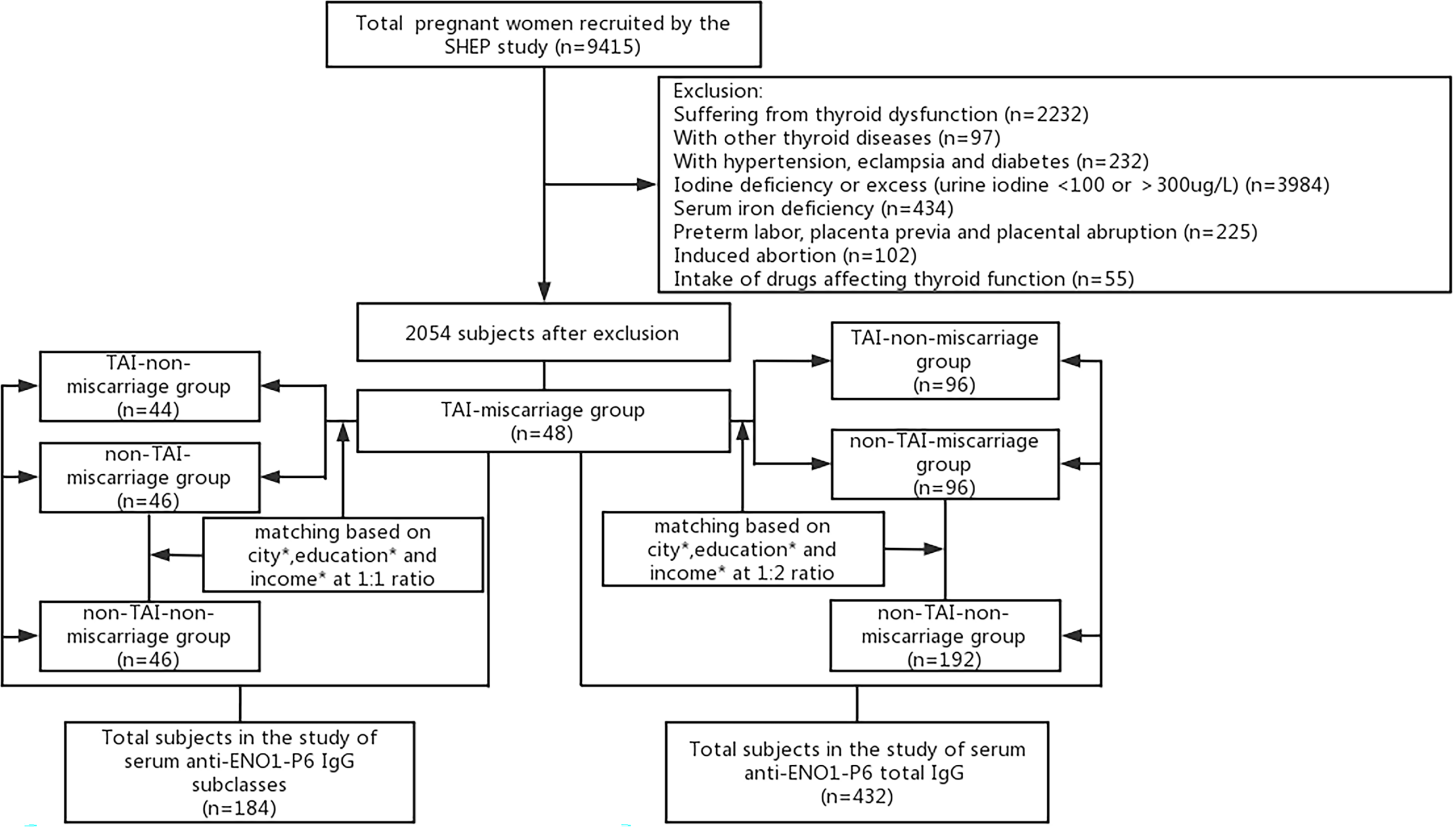
Flowchart of the inclusion and exclusion of participants in this study. *Residential city, educational background, family income.

The inclusion criteria for this study were as follows: residing in the same region for at least 5 years; age 19–45 years; during the 4–12 weeks of pregnancy; urine iodine at 100–300 g/L (relatively sufficient iodine nutrition) (30); serum ferritin (SF) of ≥20 μg/L (no iron deficiency) (31), serum free thyroxine (FT4) and TSH within the trimester-specific reference ranges of 12.35–20.71 pmol/L and 0.14–4.87 mIU/L, respectively, according to our previous study (32). The exclusion criteria for this study were as follows: participants with thyroid diseases except TAI; intake of any drugs affecting thyroid functions; with a known personal history of inherited, other chronic or autoimmune inflammatory diseases (e.g., diabetes, hypertension, and antiphospholipid syndrome); with placenta previa, preterm labor, and placental abruption during pregnancy; or undergoing artificial abortion. The pregnancy outcome focused on by this study was miscarriage, which either occurred during this pregnancy or in the last 5 years. Following the inclusion and exclusion criteria, a total of 48 TAI women who experienced spontaneous pregnancy loss were eventually recruited into this study as the TAI-miscarriage group. The other three following groups were acquired through 1:2 or 1:1 random sampling using propensity score matching (PSM) for residential city, educational background, and family income based on the TAI-miscarriage group, respectively (**Figure 1**). The serum samples left were not enough for the examination of IgG subclasses in some participants, so 1:1 random sampling was performed. The TAI-non-miscarriage group referred to TAI women without pregnancy loss (N = 96 for anti-ENO1-P6 total IgG, and N = 44 for its IgG subclasses). Non-TAI-miscarriage group consisted of non-TAI women complicated by spontaneous pregnancy loss (N = 96 for anti-ENO1-P6 total IgG, and N = 46 for its IgG subclasses). The non-TAI-non-miscarriage group was made of non-TAI women without pregnancy loss (N = 192 for anti-ENO1-P6 total IgG, N = 46 for its IgG subclasses). Finally, anti-ENO1-P6 total IgG was investigated in 432 euthyroid women, while anti-ENO1-P6 IgG subclasses were analyzed in 184 euthyroid women. Among the TAI-miscarriage women for the analysis of anti-ENO1-P6 total IgG, 10.4% of females underwent a second-trimester miscarriage while the others had spontaneous abortions only in the first trimester. The TAI-miscarriage women for the analysis of anti-ENO1-P6 IgG subclasses were the same as those for the total IgG. Among the non-TAI women for the analysis of anti-ENO1-P6 total IgG, 12.5% experienced a miscarriage in the second trimester, while the others underwent pregnancy losses only in the first trimester. Among those non-TAI women for anti-ENO1-P6 IgG subclass analysis, 8.7% experienced a miscarriage in the second trimester, while the others underwent pregnancy losses only in the first trimester.

### Sample Collection and Laboratory Analysis

During the early pregnancy, blood and urine samples from each subject were collected in the morning following an overnight fast. The specimens were kept at −80°C until further examination. The serum FT4, TSH, TgAb, TPOAb, and SF levels, and urinary creatinine concentration were determined using an electrochemiluminescence immunoassay with the Cobas Elesys 601 (Roche Diagnostics, Switzerland) (32, 33). The ammonium persulfate method, based on the Sandell–Kolthoff reaction, was used to detect urinary iodine concentration (33). The ratio of urinary iodine to creatinine (UI/Cr) was then calculated.

### Determination of ENO1-P6Abs and PDIA3Abs by ELISA

The serum anti-ENO1-P6 total IgG and its 4 subclass levels were measured by ELISA as previously described with slight modification (18). The recombinant ENO1-P6 peptide (FMILPVGAANFREAMR) was synthesized by Sangon Biotech (Shanghai, China). The microtiter plates were coated with recombinant ENO1-P6 peptide at either 10 ng per well for the examination of total IgG or 50 ng per well for the test of IgG subclasses. After incubation at 4°C overnight, the plates were further blocked with 1% bovine serum albumin (Sigma-Aldrich). Then they were incubated with the serum samples at a 1:250 dilution for total IgG measurement and at 1:50 for IgG subclasses for 2 h at 37 °C. After washing, the microtiter plates were further incubated with the HRP-labeled goat anti-human total IgG (Bioss, China) or HRP-labeled mouse anti-human IgG subclasses (Beijing Bersee, China), which were all diluted at 1:5,000. The enzymatic reaction was examined with TMB and terminated by hydrochloric acid. Finally, the absorbance values were measured at 450 nm using a microplate reader (Bio-Rad 680, Bio-Rad, CA, USA). The intra-assay coefficients of variation (CVs) were 2.7–9.5% (total IgG), 1.7–2.2% (IgG1), 1.2–5.8% (IgG2), 5.6–6.6% (IgG3), and 9.2–11.8% (IgG4), and the inter-assay CVs were 4.5–16.2% (total IgG), 0.2–11.3% (IgG1), 5.9–13.3% (IgG2), 0.3–10.9% (IgG3), and 6.3–11.9% (IgG4), respectively. The serum PDIA3Ab levels have been determined using ELISA as described in our previous study (14).

### Statistical Analysis

All statistical analyses were performed using SPSS software (version 23.0, IBM Corporation, NY, USA) and the R statistical programming language. The Kruskal–Wallis test with *post-hoc* Bonferroni correction was adopted for the comparisons among the 4 groups, while the Mann–Whitney *U* test was used for the comparison between 2 groups. The frequencies of categorical variables were compared using the chi-square test (or Fisher’s exact test if required). The relationships between anti-ENO1-P6 total IgG and serum FT4, TSH, TPOAb, and TgAb levels were assessed by Spearman correlation analysis (SCA). Binary logistic regression analysis (LRA) was used to evaluate the independent risk variables for miscarriage. Receiver operating characteristic (ROC) curve analysis was used to determine the optimal cut-off values for serum ENO1-P6Abs. The trend test was used to examine the titer-dependent associations between serum TPOAb, TgAb, and ENO1-P6Ab levels and miscarriage prevalence in euthyroid TAI women. Logistic regression was used to estimate the multiplicative interaction. Statistical significance was determined by *P <*0.05. The calculation of the relative excess risk due to interaction (RERI), attributable proportion due to interaction (AP), and synergy index (SI) through the R package was used to analyze the additive interaction (34).

## Results

### Baseline Characteristics of Subjects

The baseline characteristics of the cohort are shown in **Tables 1**, **2**. There were no significant differences in age, BMI, serum FT4, and SF levels (**Table 1**). Among the euthyroid non-TAI women, the percentage of those with a history of smoking, drinking and the serum TSH level were significantly higher, and the gestational age was markedly lower in the ones with than without miscarriage (**Table 1**). Nonetheless, they were not significantly different between the miscarriage and non-miscarriage groups with TAI. Among the groups of subjects enrolled in the study of anti-ENO1-P6 IgG subclasses, only the gestational age was pronouncedly lower in the non-TAI women with than in women without miscarriage (**Table 2**).

**Table 1:** Baseline characteristics of participants in the study of serum anti-ENO1-P6 total IgG.

	euthyroid women without TAI (non-TAI)		euthyroid women with TAI		*P*-value			
					Non-miscarriage	Miscarriage	Non-TAI	TAI
	non-miscarriage group(n = 192)	miscarriage group(n = 96)	non-miscarriage group(n = 96)	miscarriage group(n = 48)	TAI *vs.* non-TAI	TAI *vs.* non-TAI	miscarriage *vs.* non-miscarriage	miscarriage *vs.* non-miscarriage
Age, years	29 (26–31)	30 (27–32)	29 (26–31)	30 (28–32)	NS	NS	NS	NS
Gestational age, weeks	7 (6–8)	6 (5–7)	7 (6–7)	6 (5–7)	NS	NS	<0.0001	NS
BMI, kg/m^2^	21.3 (19.5–23.2)	21.5 (20–23.4)	21.3 (19.6–23.8)	21.5 (19.8–23.2)	NS	NS	NS	NS
Smoking (%)	0 (0/192)	4.2 (4/96)	1.0 (1/96)	2.1 (1/48)	NS	NS	0.012	NS
Alcohol use (%)	0 (0/192)	9.4 (9/96)	2.1 (2/96)	2.1 (1/48)	NS	NS	<0.0001	NS
TSH, mIU/L	1.62 (0.94–2.37)	2.01 (1.54–2.62)	2.01 (1.21–2.50)	2.24 (1.43–3.07)	NS	NS	0.016	NS
FT4, pmol/L	16.59 (15.36–17.61)	16.35 (15.19–17.62)	16.45 (15.18–17.86)	16.28 (15.15–17.38)	NS	NS	NS	NS
TPOAb, IU/ml	7.92 (5.00–11.48)	6.77 (5.00–9.68)	15.77 (6.87–112.03)	32.88 (8.21–102.78)	<0.0001	<0.0001	NS	NS
TgAb, IU/ml	15.64 (12.00–24.65)	15.99 (12.13–21.70)	209.90 (136.48–349.38)	194.40 (119.60–336.48)	<0.0001	<0.0001	NS	NS
Serum ferritin, μg/L	70.29 (50.50–105.80)	83.16 (57.28–121.13)	65.11(45.66-92.66)	75.76 (50.60–100.15)	NS	NS	NS	NS
Urinary iodine/creatinine, μg/g	101.40 (78.66–140.12)	108.47 (84.78–141.00)	92.34 (64.49–117.12)	105.65 (83.90–141.79)	0.032	NS	NS	NS

The above information, samples of serum and urine were collected from the pregnant women in the first trimester when they were enrolled into the SHEP study. The above data are presented as median (25-75th percentiles) for continuous variables or as n (%) for categorical variables.

P-value lower than (0.05/4 = 0.0125) is considered statistically significant after corrected for multiple comparisons using chi-square test (or Fisher’s exact test if required).

P-value lower than 0.05 is considered statistically significant after corrected for multiple comparisons using Kruskal–Wallis test followed by post-hoc Bonferroni test.

NS, non-significant.

**Table 2:** Baseline characteristics of participants in the study of serum anti-ENO1-P6 IgG subclasses.

	euthyroid women without TAI (non-TAI)		euthyroid women with TAI		*P* value			
					Non-miscarriage	Miscarriage	Non-TAI	TAI
	non-miscarriage group(n = 46)	miscarriage group(n = 46)	non-miscarriage group(n = 44)	miscarriage group(n = 48)	TAI *vs.* non-TAI	TAI *vs.* non-TAI	miscarriage *vs.* non-miscarriage	miscarriage *vs.* non-miscarriage
Age, years	28 (26–30)	29 (27–34)	28 (26–32)	30 (28–32)	NS	NS	NS	NS
Gestational age, weeks	7 (6–8)	6 (5–7)	7 (5–7)	6 (5–7)	NS	NS	0.022	NS
BMI, kg/m^2^	21.2 (19.4–22.8)	21.1 (19.4–23.2)	21.7 (19.8–23.9)	21.5 (19.8–23.2)	NS	NS	NS	NS
Smoking (%)	2.2 (1/46)	2.2 (1/46)	2.3 (1/44)	2.1 (1/48)	NS	NS	NS	NS
Alcohol use (%)	0 (0/46)	8.7 (4/46)	2.3 (1/44)	2.1 (1/48)	NS	NS	NS	NS
TSH, mIU/L	1.87 (1.19–2.40)	1.90 (1.47–2.61)	2.11 (1.49–2.51)	2.24 (1.43–3.07)	NS	NS	NS	NS
FT4, pmol/L	16.73 (15.61–17.73)	15.92 (15.05–16.95)	15.95 (15.18–17.67)	16.28 (15.15–17.38)	NS	NS	NS	NS
TPOAb, IU/ml	7.49 (5.00–12.36)	6.77 (5.08–10.17)	11.78 (5.00–51.48)	32.88 (8.21–102.78)	NS	<0.0001	NS	NS
TgAb, IU/ml	17.03 (12.01–23.39)	16.53 (12.37–23.38)	176.40 (110.00–307.75)	194.40 (119.60–336.48)	<0.0001	<0.0001	NS	NS
Serum ferritin, μg/L	71.40 (50.54–120.65)	76.53 (56.89–107.23)	66.13 (51.95–98.45)	75.76 (50.60–100.15)	NS	NS	NS	NS
Urinary iodine/creatinine, μg/g	104.90 (71.96–135.43)	112.02 (85.39–138.58)	93.74 (75.23–120.24)	105.65 (83.90–141.79)	NS	NS	NS	NS

The above information, samples of serum and urine were collected from the pregnant women in the first trimester when they were enrolled into the SHEP study. The above data are presented as median (25-75th percentiles) for continuous variables or as n (%) for categorical variables.

P-value lower than (0.05/4 = 0.0125) is considered statistically significant after corrected for multiple comparisons using chi-square test (or Fisher’s exact test if required).

P-value lower than 0.05 is considered statistically significant after corrected for multiple comparisons using Kruskal-Wallis test with post-hoc Bonferroni test.

NS, non-significant.

### Comparisons of Serum ENO1-P6Abs

**Figure 2:**
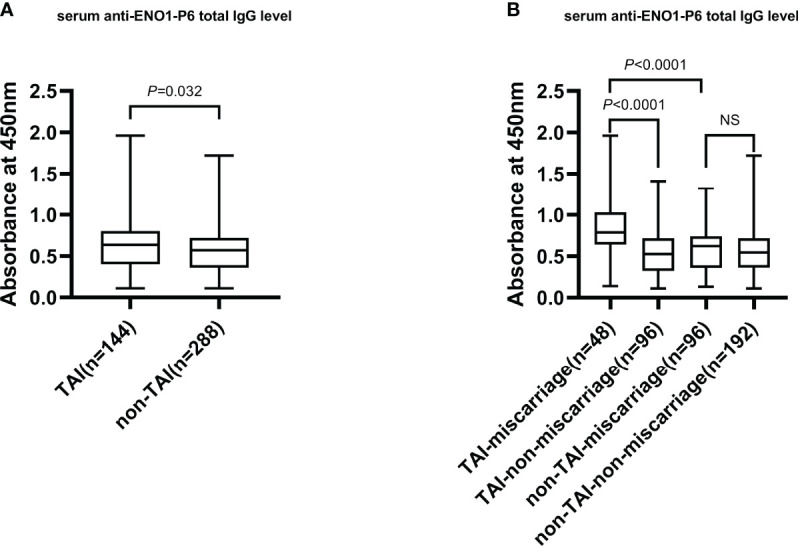
The serum total IgG level against ENO1-P6 epitope in all participants. The serum level of anti-ENO1-P6 total IgG was measured by ELISA, and reflected by the absorbance at 450 nm. Box plots are used to show the data, with boxes representing the 25th and 75th percentiles, bars representing the medians, and whiskers representing the minimum and maximum values. The Mann–Whitney U test **(A)** and the Kruskal–Wallis test followed by Bonferroni *post-hoc* analysis **(B)** were used to compare the results between the groups. NS, non-significant.

**Figure 3:**
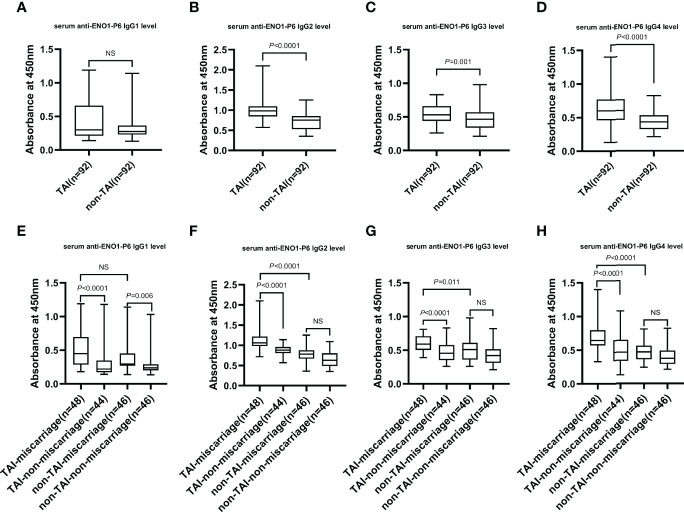
The serum levels of IgG subclasses against the ENO1-P6 epitope in all the participants. Serum anti-ENO1-P6 IgG subclass levels were determined by ELISA and reflected by the absorbance at 450 nm. Box plots are used to show the data, with boxes representing the 25th and 75th percentiles, bars representing the medians, and whiskers representing the minimum and maximum values. The Mann–Whitney U test **(A–D)** and the Kruskal-Wallis test followed by Bonferroni *post-hoc* analysis **(E–H)** were used to compare the results between the different groups. NS, non-significant.

The serum anti-ENO1-P6 total IgG, IgG2, IgG3, and IgG4 subclass levels were significantly higher in euthyroid TAI females than non-TAI ones. Additionally, the serum anti-ENO1-P6 total IgG and its 4 subclass levels were markedly raised, especially IgG2, in the TAI-miscarriage group compared with those of the TAI-non-miscarriage group. Furthermore, they were markedly higher in the TAI-miscarriage group than the non-TAI miscarriage group except serum anti-ENO1-P6 IgG1 level. Interestingly, only the latter was significantly higher in non-TAI women with miscarriage than in women without miscarriage. These findings indicate that the highly expressed ENO1-P6Abs in the serum were associated with the development of TAI-related miscarriage (**Figures 2**, **3**).

### Correlations of Anti-ENO1-P6 Total IgG With FT4, TSH, TPOAb, and TgAb in the Sera of Euthyroid TAI Women

The associations between serum FT4, TSH, TPOAb, TgAb, and anti-ENO1-P6 total IgG in euthyroid TAI women with and without pregnancy loss were analyzed by SCA, respectively. There was a weak correlation between serum anti-ENO1-P6 total IgG and TPOAb levels in TAI females with pregnancy loss but not in those without miscarriage (**Table 3**). These findings suggest that the predictive role of TPOAb expression in the occurrence of pregnancy loss reported in previous studies may be due to the concurrent existence of ENO1-P6Abs.

**Table 3:** Spearman correlation analysis of anti-ENO1-P6 total IgG expression with the levels of FT4, TSH, TPOAb, and TgAb among euthyroid TAI women.

Anti-ENO1-P6 total IgG	FT4	TSH	TPOAb	TgAb
TAI-miscarriage group	r_s_ = −0.221	r_s_ = −0.070	r_s_ = 0.302	r_s_ = 0.166
(n = 48)	NS	NS	*P* = 0.037	NS
TAI-non-miscarriage group	r_s_ = −0.027	r_s_ = −0.028	r_s_ = 0.110	r_s_ = −0.052
(n = 96)	NS	NS	NS	NS

Serum level of anti-ENO1-P6 total IgG was determined by ELISA and reflected by the absorbance at 450 nm.

NS, non-significant.

### Investigation of Independent Risk Factors for Miscarriage Among Euthyroid Women

**Figure 4:**
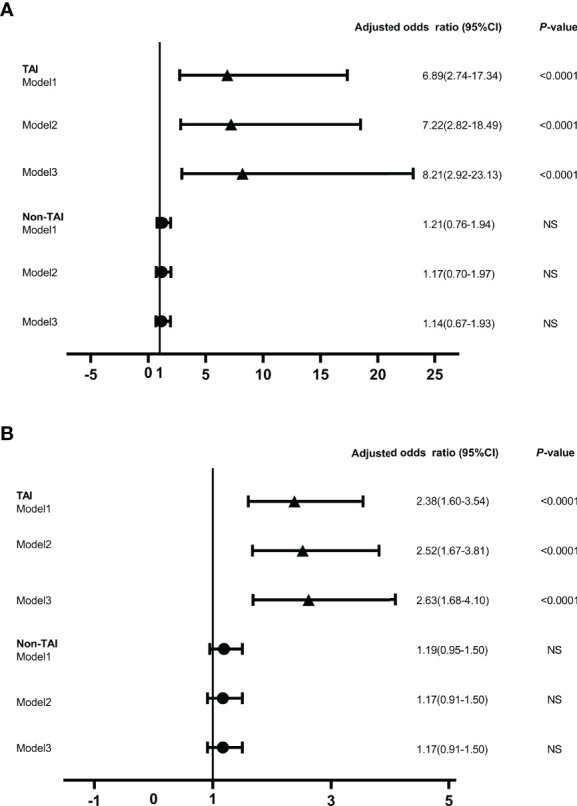
The odds ratios of serum anti-ENO1-P6 total IgG level associated with miscarriage in the euthyroid TAI females (n = 144) and non-TAI females (n = 288). Binary logistic regression analysis was conducted in Models 1–3 following the adjustment of the potential covariates. NS, non-significant. Model 1. Age was adjusted. Model 2. Age, BMI and gestational age were adjusted. Model 3. Age, BMI, gestational age, UI/Cr, serum ferritin, TSH, FT4, TPOAb, and TgAb levels (only in euthyroid TAI women) were adjusted. The serum level of anti-ENO1-P6 total IgG was included in the binary logistic regression analysis after natural logarithm-transformation **(A)** and quartile stratifications of absorbance at 450 nm **(B)**.

**Figure 5:**
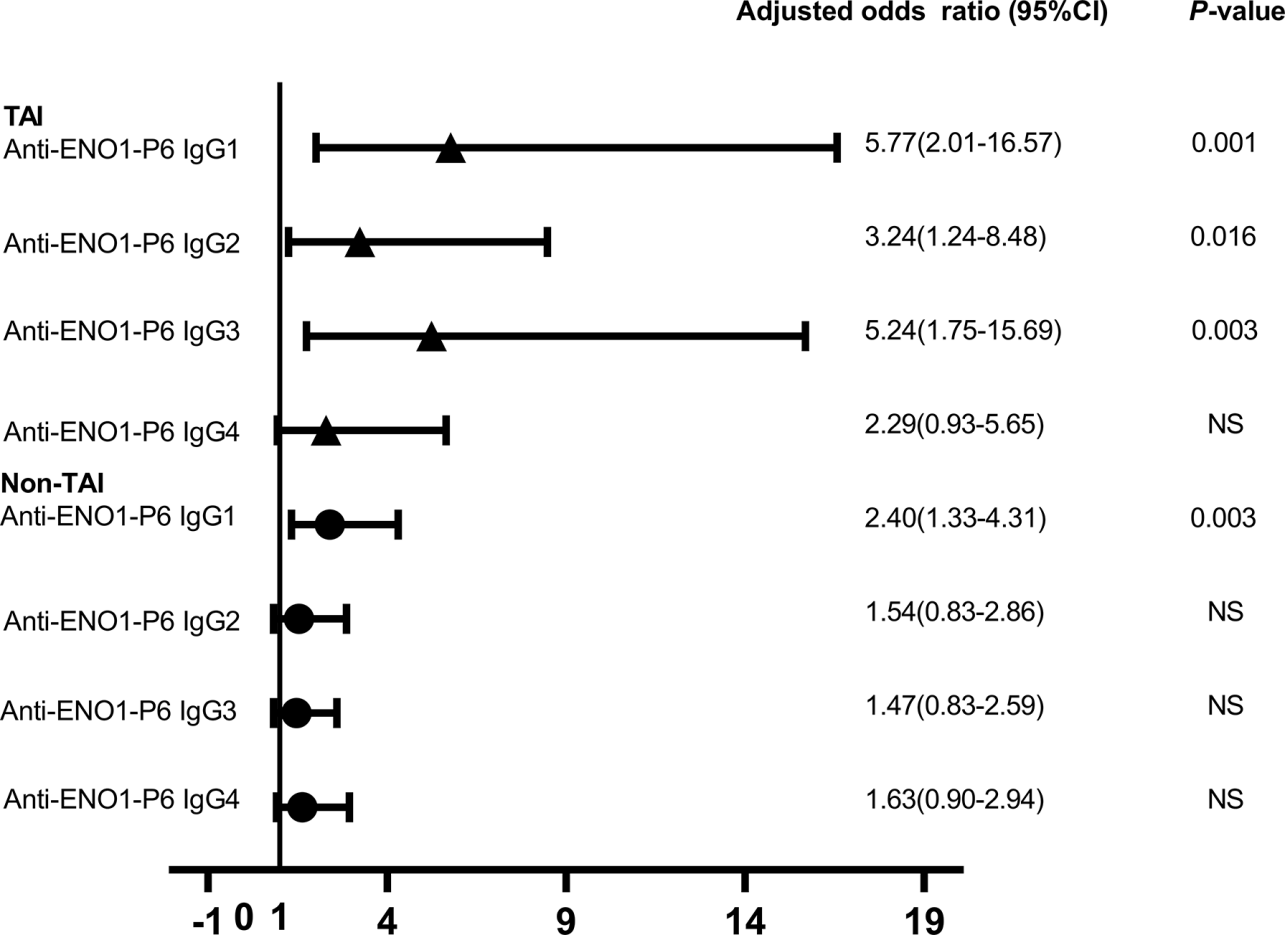
The odds ratios of serum anti-ENO1-P6 IgG subclass levels associated with the miscarriage in the euthyroid TAI females (n = 92) and those non-TAI females (n = 92). Binary logistic regression analysis was performed after adjusting for age, BMI, gestational age, UI/Cr, serum ferritin, TSH, FT4, TPOAb, and TgAb levels (only in euthyroid TAI women). NS, non-significant. Serum levels of anti-ENO1-P6 IgG subclasses after quartile stratifications of absorbance at 450 nm were included in the logistic regression analysis.

Increased maternal age, obesity, iron deficiency, thyroid dysfunction, abnormal iodine nutritional status, smoking and drinking have been reported as potential risk factors for miscarriage in previous studies (6, 31, 35–37). In this study, the number of subjects with a smoking and alcohol use history were both too small to be included in the LRA. When ENO1-P6Ab expression was not included, the LRA showed that age, BMI, gestational age, UI/Cr, serum FT4, TSH, SF, TPOAb, and TgAb levels were not independently related to pregnancy loss in those euthyroid TAI females (**Table 4**). However, both the older age and the smaller gestational age were related to a raised risk of pregnancy loss in the euthyroid non-TAI females (**Table 5**). To investigate the independent relationships between the expression of ENO1-P6Abs and miscarriage among euthyroid TAI and non-TAI females, serum anti-ENO1-P6 total IgG was further included in Models 1–3 of LRA after natural logarithm-transformation and quartile stratification of the absorbance at 450 nm, respectively (**Figure 4**). Afterwards, serum levels of anti-ENO1-P6 IgG1-4 were included and analyzed in the LRA after quartile stratification of the absorbances at 450 nm (**Figure 5**). Not only the upraised serum total IgG but also the IgG1, IgG2, and IgG3 subclasses against ENO1-P6 were independently associated with an increased risk of pregnancy loss in euthyroid TAI females, while high serum anti-ENO1-P6 IgG1 expression was still independently related to a raised risk of miscarriage in euthyroid women without TAI. These findings further indicate that the expression of ENO1-P6Abs is an important and independent risk factor for pregnancy loss among euthyroid TAI females.

**Table 4:** Binary logistic regression analysis of the potential risk factors for miscarriage without ENO1-P6Abs included among the euthyroid TAI women.

	Model 1		Model 2		Model 3	
	OR value(95% CI)	*P*-value	OR value (95% CI)	*P*-value	OR value (95% CI)	*P*-value
Age, years	1.11 (1.00–1.23)	0.042	1.13 (1.01–1.26)	0.034	1.11 (0.99–1.25)	NS
Gestational age, weeks	ND	ND	0.77 (0.57–1.03)	NS	0.77 (0.57–1.05)	NS
BMI, kg/m^2^	ND	ND	1.01 (0.89–1.16)	NS	0.95 (0.83–1.10)	NS
Serum ferritin, μg/L	ND	ND	ND	ND	1.00 (1.00–1.01)	NS
Urinary iodine/creatinine, μg/g	ND	ND	ND	ND	1.01 (1.00–1.02)	NS
TSH, mIU/L	ND	ND	ND	ND	1.31 (0.82–2.10)	NS
FT4, pmol/L	ND	ND	ND	ND	0.88 (0.66–1.16)	NS
TPOAb, IU/ml	ND	ND	ND	ND	1.00 (0.997–1.00)	NS
TgAb, IU/ml	ND	ND	ND	ND	1.00 (0.997–1.00)	NS
Alcohol use	ND	ND	N/A	N/A	N/A	N/A
Smoking	ND	ND	N/A	N/A	N/A	N/A

In Model 1, only age was included for analysis.

In Model 2, age, BMI and gestational age were included for analysis.

In Model 3, all those potential risk factors were included, which consisted of age, BMI, gestational age, serum ferritin, urinary iodine/creatinine as well as serum levels of TSH, FT4, TPOAb, and TgAb.

ND, not done in this model.

N/A, not applicable, which was due to only two participants with smoking and three participants with alcohol use.

NS, non-significant.

**Table 5:** Binary logistic regression analysis of the potential risk factors for miscarriage without ENO1-P6Abs included among the euthyroid women without TAI. .

	Model 1		Model 2		Model 3	
	OR value(95% CI)	*P*-value	OR value (95% CI)	*P*-value	OR value (95% CI)	*P*-value
Age, years	1.09 (1.02–1.17)	0.016	1.11 (1.03–1.20)	0.006	1.11 (1.03–1.20)	0.008
Gestational age, weeks	ND	ND	0.48 (0.37–0.63)	<0.0001	0.49 (0.38–0.64)	<0.0001
BMI, kg/m^2^	ND	ND	0.97 (0.90–1.04)	NS	0.97 (0.90–1.04)	NS
Serum ferritin, μg/L	ND	ND	ND	ND	1.00 (0.997-1.01)	NS
Urinary iodine/creatinine, μg/g	ND	ND	ND	ND	1.00 (0.995-1.00)	NS
TSH, mIU/L	ND	ND	ND	ND	1.23 (0.89-1.69)	NS
FT4, pmol/L	ND	ND	ND	ND	0.99 (0.83-1.17)	NS
Alcohol use	ND	ND	N/A	N/A	N/A	N/A
Smoking	ND	ND	N/A	N/A	N/A	N/A

In Model 1, only age was included for analysis.

In Model 2, age, BMI and gestational age use were included for analysis.

In Model 3, all those potential risk factors were included, which consisted of age, BMI, gestational age, serum ferritin, urinary iodine/creatinine as well as serum levels of TSH and FT4.

ND, not done in this model.

N/A, not applicable, which was due to only four participants with smoking and nine participants with alcohol use.

NS, non-significant.

### The Predictive Value of Serum ENO1-P6Abs in Euthyroid TAI Women

**Figure 6:**
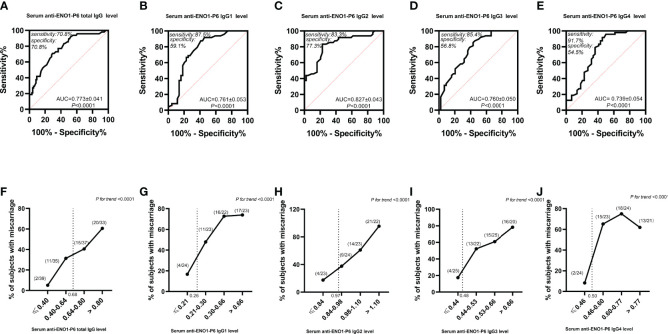
Relationship between anti-ENO1-P6 total IgG and IgG1, IgG2, IgG3, and IgG4 subclass expressions in the serum and the development of miscarriage in euthyroid TAI females. The ROC curve of the anti-ENO1-P6 total IgG and IgG1, IgG2, IgG3, and IgG4 subclass expressions for miscarriage prediction **(A–E)**, and the titer-dependent association between serum anti-ENO1-P6 total IgG and IgG1, IgG2, IgG3, and IgG4 subclass levels after quartile stratifications and the prevalence of miscarriage **(F–J)**. NS, non-significant. The serum levels of ENO1-P6Abs were determined by ELISA, and reflected by the absorbance at 450 nm.

**Figure 7:**
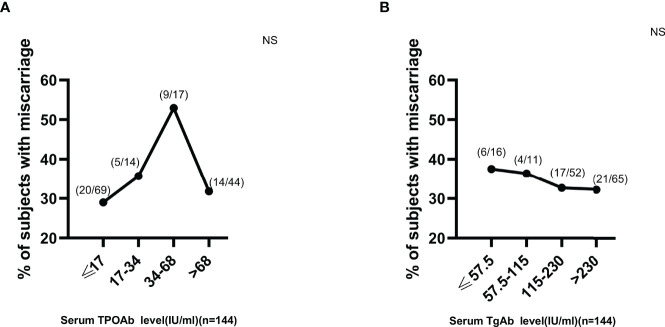
No titer-dependent effect of either serum TPOAb or TgAb expression on miscarriage development in euthyroid TAI females. NS, non-significant. The manufacturer (Roche Diagnostics, Switzerland) provided the reference ranges for serum TPOAb (≤34 IU/ml) and TgAb (≤115 IU/ml).

Based on the above findings, ROC curves and titer-dependence trend analysis were used to further explore the predictive values of serum ENO1-P6Ab expressions in the occurrence of pregnancy loss among euthyroid TAI females. The area under the ROC curve (AUC) of anti-ENO1-P6 IgG2 was the largest (0.827 ± 0.043, **Figure 6**). Based on the largest Youden index (0.416, 0.466, 0.606, and 0.422), the corresponding cut-off OD450 values of serum ENO1-P6Abs were 0.68 (total IgG), 0.26 (IgG1), 0.97 (IgG2), and 0.48 (IgG3), respectively (**Figure 6**). After adjusting for age, gestational age, BMI, UI/Cr, serum FT4, TSH, SF, TPOAb, and TgAb levels, it was determined that each 0.1 OD value increase in serum anti-ENO1-P6 total IgG, IgG1, IgG2, and IgG3 subclass levels above the corresponding cut-off values (0.68, 0.26, 0.97, and 0.48) was associated with the 45.4% (total IgG), 19.5% (IgG1), 307% (IgG2), and 221% (IgG3) higher risks of pregnancy loss in euthyroid TAI females, respectively. Additionally, each of those Ab levels was classified into 4 grades for titer-dependence trend analysis based on either the quartiles or upper limits of reference ranges. The prevalence of miscarriage was significantly raised with the increased serum anti-ENO1-P6 total IgG, IgG1, IgG2, IgG3, and IgG4 levels in euthyroid TAI women (**Figures 6F–J**). However, it did not show a titer-dependent change with the increase in serum TPOAb or TgAb levels (**Figure 7**). The above results indicate that serum ENO1-P6Abs can become important prediction factors for the occurrence of pregnancy loss in euthyroid TAI females, and especially its IgG2 subclass, may exhibit optimistic sensitivity and specificity. Interestingly, when the anti-ENO1-P6 IgG4 subclass was separately analyzed, the *P-*values of the ROC and titer-dependent trend analyses were both less than 0.05 (**Figure 6**). However, when all the four IgG subclasses were included in the LRA, anti-ENO1-P6 IgG4 was not an independent risk factor for miscarriage in euthyroid TAI females (**Figure 5**).

### The Correlation of Serum Anti-ENO1-P6 and Anti-PDIA3 Total IgGs in Euthyroid TAI Women

We have recently found that serum PDIA3Ab expression was also independently associated with TAI-related miscarriage (14). It was further investigated whether there was any interaction between anti-ENO1-P6 and anti-PDIA3 total IgG expressions in TAI-related miscarriage. Thirty-six TAI women with miscarriage and 66 matched TAI women without miscarriage who had been tested for both the two Abs in the sera were recruited in this pilot study. Serum anti-ENO1-P6 and anti-PDIA3 total IgGs were both included with the above potential risk factors in Models 1–3 of LRA after natural logarithm-transformation. Both of them were independent risk factors for spontaneous abortion in euthyroid TAI females (**Supplementary Table 1**). According to corresponding cut-off values of anti-ENO1-P6 and anti-PDIA3 total IgGs (14), the serum levels of two Abs were converted to binary variables. The binary variables were used to analyze interactions. No multiplicative interaction between anti-ENO1-P6 and anti-PDIA3 total IgGs was observed (OR = 2.27, 95% CI = 0.26, 19.88, *P* = 0.458). However, there was an additive interaction between anti-ENO1-P6 and anti-PDIA3 total IgGs on the development of miscarriage (RERI = 23.6, 95% CI = −18.1, 65.3; AP = 0.79, 95% CI = 0.47, 1.11; SI = 5.37, 95% CI = 1.03, 27.87). SCA was used to analyze the association between the serum expressions of anti-ENO1-P6 and anti-PDIA3 total IgGs among euthyroid TAI females with and without pregnancy loss. The results showed that there was a positive correlation between anti-ENO1-P6 and anti-PDIA3 total IgG levels in the TAI-miscarriage group (*P =* 0.027; r_s_ = 0.368), but not in the TAI-non-miscarriage group (*P =* 0.317; r_s_ = 0.125). These results indicate that both ENO1-P6Ab and PDIA3Ab are independent risk factors for pregnancy loss among euthyroid TAI women. Our previous studies have suggested that they may be involved in the development of extrathyroidal damage (e.g., encephalopathy) through different mechanisms in TAI individuals (18, 38), which needs to be further investigated for the occurrence of miscarriage.

## Discussion

TAI is the most prevalent autoimmune disorder, affecting up to 20.35% of women (2). There was a 2–3 times higher prevalence rate of pregnancy loss among euthyroid TAI women than non-TAI women (5). However, it is still unknown why TAI women are more prone to miscarriage. TPOAb and TgAb are important markers for the existence of TAI. Some studies have examined the relationship between the expression of ATA and pregnancy loss among TAI females. However, the direct and independent relationship between the serum ATA levels and the occurrence of pregnancy loss has not been demonstrated yet (4, 5, 11, 39–43). In a previous study, LRA indicated that TPOAb positivity was not associated with a raised risk of poor pregnancy outcomes in euthyroid women (41). The current study also systematically investigated the potential risk factors for pregnancy loss among euthyroid TAI women, namely, age, gestational age, BMI, UI/Cr, SF, serum TSH, FT4, TPOAb, and TgAb levels. Only age and gestational age were independently related to the occurrence of pregnancy loss in euthyroid non-TAI women. There was no independent relationship between the risk of pregnancy loss and the above factors among euthyroid TAI women. In previous studies, no titer-dependent association was found between the serum levels of ATA and pregnancy loss (4, 11). This study did not show any independent correlation of serum TPOAb or TgAb expression with the occurrence of miscarriage or any titer-dependent relationship between them in euthyroid TAI women.

There may be complex mechanisms for TAI-related miscarriage. Although the specific mechanisms have not been clearly known yet, the abnormal activation of the maternal immune system may result in pregnancy loss (9, 10, 44). NOSA has received much attention besides T, NK cell abnormalities, and concurrent autoimmune diseases (9, 45). Liu et al. found no significant difference in the proportions of peripheral CD3^+^T, CD4^+^T, CD8^+^T, NK, and B cells between recurrent miscarriage patients with and without TAI (46). The findings indicate that there may be a mild correlation between abnormal cellular immunity and TAI-related miscarriage. It has been reported that euthyroid TAI women have one or more NOSA expressed in their sera (47). One of the important NOSA related to miscarriage is antiphospholipid Abs (aPLs) (48). APLs are a family of autoantibodies that recognize various combinations of phospholipids such as cardiolipin, phosphatidylserine, phosphatidylethanolamine, and phospholipid-binding proteins. The latter mainly include β2 glycoprotein-I, prothrombin or annexin-V, and protein-Z. These Abs have been linked to a possible contribution to reproductive failure (49). Anticardiolipin Abs, anti-β2-glycoprotein I Abs, and lupus anticoagulant are usually determined to screen for antiphospholipid syndrome (50). However, aPLs have been found to explain only a tiny part of the occurrence of miscarriage in patients with polyautoimmunity (51). Besides, another study has reported that patients with aPLs alone had higher percentages of spontaneous pregnancies and live births when compared with those positive for anti-thyroid antibodies alone or with aPLs (52). Antinuclear Abs (ANAs) are indeed common in NOSA under the conditions of many autoimmune diseases. The prevalence of ANA by indirect immunofluorescence has been reported as 5.92% in China (53). The frequency of serum ANA positivity has been found to be significantly higher in the patients with recurrent miscarriage (20.6%) than in the control females (6.7%) (54). One study has compared the prevalence of NOSA (ANA, antiphospholipid antibodies, anti-dsDNA, anti-ssDNA, anti-histone, and anti-Scl70 autoantibodies) between TAI and non-TAI women, who all presented a history of recurrent pregnancy loss or unexplained infertility (47). The total prevalence of anti-dsDNA, anti-ssDNA, anti-histone, and anti-Scl70 was significantly higher in women with TAI (35.2%) than in those without TAI (11.4%). However, there was no significant difference in the positive prevalence of either aPLs or ANA between women with TAI (aPLs 46.3%, ANA 29.6%) and those without TAI (aPLs 43.6%, ANA 28.0%) (47). Another study has found that the positivity rate of thyroid Abs in women with recurrent miscarriage was increased, while the expression of ANA was not detectable (55). Both studies have indicated that ANA may not be the main specific factor contributing to TAI-related miscarriage. Although we have recently found that PDIA3Ab may be involved in TAI-related miscarriage, there are rare studies on the relationships between NOSA expressions and this disease. In this investigation, we focused on the role of another NOSA in the development of TAI-related miscarriage.

ENO1 is a multifunctional protein located in the cellular nucleus, cytoplasm, and surface that plays a vital role in glucose metabolism and inflammatory responses (15, 16, 56). ENO1 has also been known as a SUPER protein and is expressed on the surface of early apoptotic cells, which can trigger an autoimmune response (15, 16, 57). ENO1Abs have been reported in various autoimmune diseases, such as Hashimoto’s encephalopathy (HE), Behcet’s disease, rheumatoid arthritis (RA), multiple sclerosis, cancer-associated retinopathy (CAR), and lupus nephritis, and its positivity prevalence in the serum is found to be about 6–83% (16, 18, 58–63). Our previous study found that ENO1 protein was expressed in thyroid tissue, and serum ENO1Ab levels were significantly increased in the mice with EAT established by only Tg immunization (18). It further indicates that ENO1Abs can be induced *in situ*, which may be due to increased thyrocyte apoptosis and/or intermolecular epitope diffusion in the development of TAI but not caused by the other co-occurrent autoimmune diseases (64, 65).

Additionally, ENO1 expression has been found in placental and decidua tissues (19, 21, 66). It was significantly decreased in the placentas of uRM patients compared with that of normal pregnant women (19). A recent study has reported that serum ENO1Abs were significantly increased in uRM patients and could inhibit the production of β-hCG and progesterone from trophoblast cells cultivated *in vitro*, which may result in the occurrence of miscarriage (21). ENO1Abs have been considered a kind of anti-endometrial Ab existing in the sera of endometriosis patients and are potentially responsible for autoimmune premature ovarian failure (22, 23). ENO1Ab expression was also found prevalently in tubal factor infertility patients and was associated with IVF implantation failure (24). All those studies suggest that ENO1Abs play an important role in the occurrence of miscarriage, and involved in TAI-related miscarriage.

Since ENO1Abs have been found in various autoimmune diseases, the DSEs need to be clarified. However, they have been investigated in only a few studies. ENO1Abs in the sera of HE patients have been shown to specifically recognize the amino-terminal epitopes (aa1-157) from ENO1 (67). The epitope aa26-40 of ENO1 was specifically recognized by the serum ENO1Abs in RA patients (59). The epitopes aa31–38, aa176–183, and aa421–428 of ENO1 were mainly recognized by ENO1Abs from both CAR patients and healthy controls, while epitope aa56–63 was recognized only by those from CAR patients (62). The epitopes recognized by ENO1Abs separated from the sera and renal biopsy samples of lupus nephritis patients have been investigated. Its IgG2 subtype specifically recognized epitope aa170–182 and its IgG4 subtype recognized aa 98–165 (58). Another two linear regions of ENO1, aa53–87 and aa207–238, were specifically recognized by ENO1Abs from endometriosis patients (22). However, the target epitopes of ENO1 specific to miscarriage due to various causes have not been investigated yet.

In this study, we have performed a series of analyses to explore whether the Abs against the epitope aa 168–183 of the ENO1 protein (P6) were involved in the development of TAI-related miscarriage. This epitope was achieved by bioinformatics analysis using the IEDB, BCpred, and Ellipro databases. We further found that the serum levels of total IgG and IgG2, IgG3, and IgG4 subclasses against ENO1-P6 epitope were markedly higher in TAI females than in non-TAI controls, and especially raised among euthyroid TAI females with miscarriage. LRA showed that either anti-ENO1-P6 total IgG or IgG1, IgG2, and IgG3 subtypes were independent risk factors for pregnancy loss among euthyroid TAI patients, whereas their IgG1 subtype was also independently associated with pregnancy loss among non-TAI women. The serum levels of anti-ENO1-P6 total IgG and IgG1, IgG2, and IgG3 subclass exhibited titer-dependent positive associations with the prevalence of pregnancy loss among euthyroid TAI women. The findings indicate that ENO1-P6Abs may play a critical role in the pathogenesis of TAI-related pregnancy loss and may become the predictive markers for TAI-related miscarriage. The ROC curve analyses suggest that serum ENO1-P6 IgG2 is an optimistic predictive marker based on its specificity and sensitivity. It was found that every 0.1 OD value higher anti-ENO1-P6 IgG2 level above the corresponding cut-off value (i.e., 0.97) was related to a 307% higher risk of miscarriage in euthyroid TAI women. Besides, serum TPOAb levels in euthyroid TAI females with pregnancy loss were indeed positively correlated with the expression of serum ENO1-P6-specific total IgG. This suggests that the raised risk of pregnancy loss among TPOAb-positive women may be due to the presence of ENO1-P6Abs besides PDIA3Ab (14).

At present, the precise biological functions of the ENO1-P6 epitope have not been fully understood. According to a previous study (25), the P6 epitope may be involved in maintaining the glycolytic enzymatic activity of ENO1. As a glycolytic enzyme, the loop (aa153–169) of ENO1 donates a proton to the phosphoryl of PGA, and the proton shared by Glu168 and Glu211 forms a hydrogen bond with the PGA hydroxyl group (25). It indicates the ENO1-P6 epitope participates in the conversion of PGA to PEP in the glycolytic pathway. It is well known that glycolysis is the main energy source to support normal placental development and successful pregnancy in the first trimester when the placenta has to endure the conditions of physiologically low oxygen (68, 69). Therefore, we infer that the direct binding of ENO1-P6Abs to the epitope may inhibit the glycolysis process, reduce the energy supply to the placenta and the embryo, and finally contribute to the occurrence of miscarriage. In addition, the increased serum anti-ENO1-P6 IgG1, IgG2, and IgG3 may bind with ENO1-P6 epitope on the trophoblasts, and cause their damages since all these 3 subclasses have strong binding and activating capacities to complement system and FcR fragments on the lymphocytes (70). Previous studies have been found that ENO1Abs damaged the thyrocytes through antibody-dependent cell-mediated cytotoxicity (ADCC) and injured the pancreatic ductal adenocarcinoma cells through complement-dependent cytotoxicity (CDC) (18, 71). Previous studies have indicated that uncontrolled complement activation may lead to fetal loss (72). Also, abnormal expression of complement regulators and antibodies to trophoblast surface antigens may be implicated in excess complement activation among females with recurrent spontaneous abortions (73). In autoimmune and inflammatory diseases, ENO1Abs may result in endothelial damage by generating immune complexes, activating the complement cascade, and inhibiting the binding of plasminogen to ENO1 so as to disrupt the intravascular and pericellular fibrinolytic systems and result in cell death (16). Bruschi et al. found that IgG2 was the predominant subtype of the ENO1Abs in the serum and glomerulus of LN patients (58). Thus, we deduce that the ENO1-P6Abs may also damage trophoblast cells through ADCC and/or CDC. The specific mechanisms will be further explored in the future, which may bring new therapeutic strategies for the occurrence of TAI-related pregnancy loss.

However, there are several limitations to this cohort study. This is a retrospective study with a relatively small number of subjects. A larger-sample prospective investigation needs to be performed in the future. The mechanism related to the production of ENO1Abs has not been known yet, which will be further investigated in our future work. Additionally, we did not explore the related mechanisms for ENO1-P6Abs to be involved in the development of TAI-related miscarriage in this study. However, we have designed a series of experimental studies for them. The ENO1Abs may interfere with the enzymatic activity and other physiological functions of ENO1 in the tissue cells and mediate cell injury *via* antibody-dependent cytotoxicity (18, 21). The placenta and decidua tissues have been collecting in the newly enrolled participants. We would use western blot and an ENO1 activity assay kit (Abcam) to measure the level and activity of ENO1 in the homogenates of those tissues in the future. It may help explain whether the ENO1-P6Abs affect miscarriage by directly inhibiting ENO1 enzymatic activity. We admit that ENO1-P6Abs are not the sole Abs involved in the pathogenesis of TAI-related pregnancy loss, since another potentially pathogenic Ab (i.e., PDIA3Ab) has been recently found (14). However, this study has shown that they are both independent risk factors for the occurrence of TAI-related miscarriage with synergistic additive effects. Our previous studies have suggested that the target autoantigens and potential mechanisms are different for ENO1Abs and PDIA3Abs (14, 38). Although this study did not directly determine some other NOSA, including ANA and aPLs, and those participants with a known personal history of other chronic or autoimmune inflammatory diseases, including ANA or aPLs positivity, had been excluded from this study, we would conduct a larger-scale prospective study to further observe the relationship between the expressions of all known NOSA, including ANA and multiple aPLs, and the occurrence of miscarriage in euthyroid TAI women in the future, so as to establish a biomarker panel for better predicting their pregnancy loss risk.

The findings from this study have revealed that the expressions of ENO1-P6Abs are important independent risk factors for pregnancy loss among euthyroid TAI women. ENO1-P6Abs, especially its IgG2 subclass, may be used as a new predictive biomarker for TAI-related miscarriage. Based on the previous studies, it can be deduced that ENO1-P6Abs may contribute to the risk of pregnancy loss in euthyroid TAI females through some complex mechanisms, potentially including interference with the glycolysis process, injury of trophoblast cells, and inhibition of pregnancy-related hormone production. Experimental studies on the potential mechanisms have been designed and will be completed in the near future. As a relatively specific antigenic epitope, ENO1-P6 and its Abs may become new targets for the intervention of TAI-related miscarriage.

## Data Availability Statement

The original contributions presented in the study are included in the article/**Supplementary Material**. Further inquiries can be directed to the corresponding author.

## Ethics Statement

The studies involving human participants were reviewed and approved by Medical Ethics Committee of China Medical University. The patients/participants provided their written informed consent to participate in this study.

## Author Contributions

XH—formal analysis, investigation, and writing-original draft. YL—methodology and investigation. HW—investigation, data curation, and project administration. WS—methodology and investigation. YL—methodology and investigation. ZS—resources, supervision, and funding acquisition. WT—resources and supervision. JL—conceptualization, methodology, writing—review & editing, supervision, and funding acquisition.

## Funding

This work was supported by the National Nature Science Foundation of China (grant numbers 81771741 and 81570709), the Distinguished Professor at Educational Department of Liaoning Province (grant number No. [2014]187), and the National Science and Technology Support Program (grant number 2014BA106B02).

## Conflict of Interest

The authors declare that the research was conducted in the absence of any commercial or financial relationships that could be construed as a potential conflict of interest.

## Publisher’s Note

All claims expressed in this article are solely those of the authors and do not necessarily represent those of their affiliated organizations, or those of the publisher, the editors and the reviewers. Any product that may be evaluated in this article, or claim that may be made by its manufacturer, is not guaranteed or endorsed by the publisher.
