# Supplementary material for: A Predictive Role of Autoantibodies Against the Epitope aa168–183 of ENO1 in the Occurrence of Miscarriage Related to Thyroid Autoimmunity
Source: Front Immunol. 2022 May 30;13:890502. doi: 10.3389/fimmu.2022.890502 (PMC9190245; doi:10.3389/fimmu.2022.890502)
Supplement: Supplementary file 1 [file Table_1.doc]

**Supplementary Table S1** Binary logistic regression analysis of the potential risk factors for miscarriage with anti-ENO1-P6 and anti-PDIA3 total IgGs included among the euthyroid TAI women.

|  | Model 1 | |  | Model 2 | |  | Model 3 | |
| --- | --- | --- | --- | --- | --- | --- | --- | --- |
|  | OR value  (95%CI) | *P* value |  | OR value (95%CI) | *P* value |  | OR value (95%CI) | *P* value |
| Age, years | 1.08(0.93-1.26) | NS |  | 1.07(0.91-1.26) | NS |  | 1.07(0.90-1.26) | NS |
| Anti-ENO1-P6 total IgG | **5.98(1.89-18.94)** | **0.002** |  | **5.98(1.86-19.16)** | **0.003** |  | **6.38(1.84-22.16)** | **0.004** |
| Anti-PDIA3 total IgG | **6.19(1.27-30.19)** | **0.024** |  | **7.23(1.34-38.95)** | **0.021** |  | **8.47(1.40-51.53)** | **0.020** |
| Gestational age, weeks | ND | ND |  | 0.87(0.60-1.27) | NS |  | 0.84(0.55-1.27) | NS |
| BMI, kg/m^2^ | ND | ND |  | 1.12(0.94-1.34) | NS |  | 1.08(0.89-1.32) | NS |
| Serum ferritin, μg/L | ND | ND |  | ND | ND |  | 1.01(1.00-1.02) | NS |
| Urinary iodine/creatinine, μg/g | ND | ND |  | ND | ND |  | 1.01(1.00-1.02) | NS |
| TSH, mIU/L | ND | ND |  | ND | ND |  | 1.17(0.62-2.20) | NS |
| FT4, pmol/L | ND | ND |  | ND | ND |  | 0.96(0.65-1.43) | NS |
| TPOAb, IU/mL | ND | ND |  | ND | ND |  | 1.00(0.99-1.01) | NS |
| TgAb, IU/mL | ND | ND |  | ND | ND |  | 1.00(0.997-1.00) | NS |
| Alcohol use | ND | ND |  | N/A | N/A |  | N/A | N/A |
| Smoking | ND | ND |  | N/A | N/A |  | N/A | N/A |

In model 1, age, serum levels of anti-ENO1-P6 total IgG and anti-PDIA3 total IgG were included for analysis.

In model 2, age, BMI, gestational age, serum levels of anti-ENO1-P6 and anti-PDIA3 total IgGs were included for analysis.

In model 3, all those potential risk factors were included, which consisted of age, BMI, gestational age, urinary iodine/creatinine as well as serum levels of serum ferritin, TSH, FT4, TPOAb, TgAb, anti-ENO1-P6 and anti-PDIA3 total IgGs.

Serum levels of anti-ENO1-P6 and anti-PDIA3 total IgGs had been included in the logistic regression analysis after natural logarithm-transformation.

ND, not done in this model.

N/A, not applicable, which was due to only one participants with smoking and three participants with alcohol use.

NS, non-significant.
